# Dissociation between Emotional Remapping of Fear and Disgust in Alexithymia

**DOI:** 10.1371/journal.pone.0140229

**Published:** 2015-10-13

**Authors:** Cristina Scarpazza, Elisabetta Làdavas, Giuseppe di Pellegrino

**Affiliations:** 1 Department of Psychology, University of Bologna, Bologna, Italy; 2 CsrNC, Center for studies and research in Cognitive Neuroscience, University of Bologna, Cesena, Italy; French National Centre for Scientific Research, FRANCE

## Abstract

There is growing evidence that individuals are able to understand others’ emotions because they “embody” them, i.e., re-experience them by activating a representation of the observed emotion within their own body. One way to study emotion embodiment is provided by a multisensory stimulation paradigm called emotional visual remapping of touch (eVRT), in which the degree of embodiment/remapping of emotions is measured as enhanced detection of near-threshold tactile stimuli on one’s own face while viewing different emotional facial expressions. Here, we measured remapping of fear and disgust in participants with low (LA) and high (HA) levels of alexithymia, a personality trait characterized by a difficulty in recognizing emotions. The results showed that fear is remapped in LA but not in HA participants, while disgust is remapped in HA but not in LA participants. To investigate the hypothesis that HA might exhibit increased responses to emotional stimuli producing a heightened physical and visceral sensations, i.e., disgust, in a second experiment we investigated participants’ interoceptive abilities and the link between interoception and emotional modulations of VRT. The results showed that participants’ disgust modulations of VRT correlated with their ability to perceive bodily signals. We suggest that the emotional profile of HA individuals on the eVRT task could be related to their abnormal tendency to be focalized on their internal bodily signals, and to experience emotions in a “physical” way. Finally, we speculated that these results in HA could be due to a enhancement of insular activity during the perception of disgusted faces.

## Introduction

An influential model of emotion processing [1,2] suggests that, during face-to-face interaction, recognizing another’s emotion depends on re-experiencing that emotion by activating a representation of it within one’s own somatosensory system, which simulates how the other individual would feel when displaying a certain facial expression [3,4]. Thus, the observed emotion is “embodied”: we understand the facial expressions, and indeed the emotions of others, by activating similar emotions in ourselves [5]. Accordingly, neuroimaging studies have revealed that recognizing a facial expression of emotion in another person and experiencing that emotion in oneself involves overlapping neural circuits [6].

One way to study emotional embodiment is provided by a multisensory stimulation paradigm called Visual Remapping of Touch (VRT). In this paradigm, viewing another person being touched enhances the perception of near-threshold tactile stimuli on the face compared with viewing the same face being approached, but not touched, by fingers [7], and activates brain regions usually recruited during tactile perception, i.e. a network of fronto-parietal areas including the somatosensory cortices, the posterior parietal cortices and the premotor cortices [8]. The VRT effect is specific to viewing a human bodily stimulus; touch on a non-human face is not remapped onto the somatosensory system of the human observer [9]. Importantly, the effect is modulated by the emotional expression of the observed face (emotional VRT, or eVRT) [10]. Specifically, the VRT effect is enhanced when observing faces expressing fear relative to neutral expressions, but happy and angry expressions do not modulate VRT [10]. Thus, the eVRT effect seems to be specific to fearful expressions.

The eVRT effect is thought to be evoked by means of a preliminary activation of somatosensory cortices when viewing fearful faces, resulting in facilitated processing of tactile information delivered to the participant’s face [10]. Indeed, fearful faces are highly salient as they might alert the observer to a potential threat that needs to be identified so that a defensive, avoidance response may be prepared. Rapid recognition of such an emotion is therefore critical from an evolutionary perspective. This could be the reason why the enhancement of VRT emerges only with fearful facial expressions [10].

A compelling question is how the mechanism of embodying emotions works in people with emotional difficulties such as alexithymia. Alexithymia is a subclinical phenomenon characterized by a difficulty in identifying one’s own and others feelings, particularly the negative ones [11,12], flattened affect and unawareness of emotions [11,12]. Alexithymia is a stable personality trait [13,14] and is considered to be a risk factor for physical illness [15,16], and psychiatric disorders [17–19].

Using the eVRT task, it has been recently shown that the expected enhancement of the VRT effect with fearful faces, which is present in participants with low levels of alexithymia (LA), is absent in participants with high levels of alexithymia (HA) [20]. HA participants are able to remap the observed touch onto their own face since they are more accurate at detecting the tactile stimuli delivered on their own face when they see a face being touched compared to being only approached. However, they do not show the specific effect of fear on VRT. Note that the absence of fear modulation in HA cannot be explained by a difficulty in directly recognizing emotional expressions, namely fear, because neither the facial expression recognition rates nor the intensity (arousal) ratings of the emotional expressions differed between the HA and LA groups. The lack of VRT enhancement with fearful faces is instead better explained by a specific deficit in remapping fearful information conveyed by others’ faces onto the self.

Like fear, disgust is a negatively valenced emotion and has been traditionally viewed as priming an avoidance response, since it serves the function of signaling ‘contaminating’ objects, behaviors or persons that are to be avoided [21–23]. Disgust protects the body by discouraging contact with contaminating substances; likewise, moral and interpersonal disgust may protect the individual’s soul and identity, discouraging the endorsement of immoral actions [21, 24, 25]. Disgust is associated with the appraisal of being too close to something revolting, or to an indigestible object or idea [26], and it is characterized by the desire to refuse contact with the offending agent [21]. From this point of view, a disgusted face could potentially have the same function as a fearful face, i.e. enhancing vigilance to detect the source of a potential threat in the environment. Considering these functional similarities, one may predict that disgusted faces would have the same effect on VRT as fearful faces; i.e. an enhancement of the VRT effect relative to neutral faces in LA but not HA participants. We will refer to this hypothesis as the functional hypothesis.

However, the physiological underpinnings of disgust might suggest a different outcome on VRT in HA participants. For example, disgust tends to activate parasympathetic responses, reducing heart rate, blood pressure, and respiration [27,28]. Conversely, fear usually swings these systems in the opposite direction by stimulating sympathetic pathways, increasing heart rate and startle reflex [29]. The facial configuration elicited by these two emotions are different as well: when subjects are exposed to a disgusting stimuli they manifest smaller visual field and decrease in nasal volume and air velocity inspiration, while the opposite pattern was found when subjects are exposed to fearful stimuli [30,31]. A further difference between fear and disgust refers to their neural basis: the emotion of fear predominantly activates the amygdala [32–36], whereas the emotion of disgust predominantly activates the insula [32,37–40]. Finally, disgust is often considered the most visceral of all the basic emotions [41], eliciting peripheral bodily changes that facilitate the protection of the body from contaminating objects [42,43], while fear enhances the vigilance to detect the source of a potential threat in the environment, thereby preparing the body to react to external threats [33]. Interestingly, alexithymic individuals are known to experience amplified visceral sensations and to rely on physical information to judge the emotional content of stimuli [44–46]. Thus, considering the above reported physiological differences between the emotions of fear and disgust [47], and that alexithymic individuals tend to enhance the normal visceral phenomena accompanying emotional arousal [45,48,49], one may predict that disgusted faces would have a different effect on VRT compared to fearful faces, i.e. an enhancement of the VRT effect relative to neutral faces in HA but not LA participants. We will refer to this second hypothesis as the physiological hypothesis.

To test these two alternative hypotheses, i.e. the functional hypothesis and the physiological hypothesis, participants with low and high levels of alexithymia took part in a tactile confrontation task [50,51]: participants were touched on the left cheek, the right cheek, or both cheeks simultaneously, and were required to report the side of stimulation. To simulate tactile extinction [52,53], stimulus intensity on one cheek was stronger than on the other cheek. In line with previous results, we predicted that, in trials of bilateral tactile stimulation, the stronger stimulus would frequently extinguish the weaker one [50,51]. While performing the tactile confrontation task, participants watched a human face with a fearful expression, a disgusted expression, or a neutral expression [54] being touched or just approached, unilaterally or bilaterally, by one or two human fingers. Different results were predicted by the functional and physiological hypotheses.

## Experiment 1

### Material and Methods

#### Participants

Three hundred university students completed the 20-item Toronto Alexithymia Scale (TAS-20; [55]. Individuals with high and low total TAS-20 scores (top quartile score >60; bottom quartile score <39) were selected in order to obtain a sample with as large a variance on alexithymia as possible. The alexithymia module of the structured interview for the Diagnostic Criteria for Psychosomatic Research (DCPR) [56], previously used in alexithymia research [57], was used to further confirm the presence or absence of alexithymia. Moreover, due to the high association between alexithymia and depression [58,59], the Italian version of the Structured Clinical Interview for DSM-IV Axis I Disorders (SCID-I; [60]), mood disorders subscale, was used to exclude participants with high levels of depression. Participants were included in the study if i) they had no history of neurological, major medical or psychiatric disorder and ii) their scores on the TAS-20 and the DPCR were congruent. Three participants with a high TAS-20 score and a low DCPR score were discarded, as well as a participant with a high TAS-20 score who reported a high level of depression on the SCID. Table 1 gives comparative information about the resulting low alexithymia (LA; n = 20) and high alexithymia (HA; n = 20) groups. All participants had equivalent educational backgrounds and were students at the University of Bologna.

**Table 1:** Demographic and alexithymia profile of low and high alexithymia groups. TAS-20: twenty-item Toronto Alexithymia Scale; DIF: difficulty in identifying feelings; DDF: difficulty in describing feelings; EOT: externally oriented thinking. SD: standard deviation. Low Alexithymia (n = 20) and High Alexithymia (n = 20) groups were obtained by selecting volunteers with low or high total scores on the TAS-20. Participants with discrepant TAS-20 and DPCR scores were excluded (cf. Material and Methods). DPCR: Alexithymia Module of Diagnostic Criteria for Psychosomatic Research scores.

	Low Alexithymia	High Alexithymia	
**n (male/female)**	20 (5/15)	20 (5/15)	
**Age, mean (SD) (years)**	22.6 (1.66)	21.8 (1.22)	
*TAS-20*	*Minimum-maximum*, *mean (SD)*	
**Total**	26–39, 35.1 (3.5)	61–77, 65.5 (5.3)	
**DIF**	8–18, 12.2 (2.6)	19–32, 24.9 (3.7)	
**DDF**	5–12, 9.1 (2.2)	16–24, 19.9 (2.4)	
**EOT**	10–16, 13.5 (2)	12–29, 20.4 (4.9)	
**DPCR**	0–2, 1.1 (0.6)	3–5, 3.6 (0.7)	

Sample size was determined a priori by conducting a power analysis using G*Power 3 [61]. A small to medium effect size (η_p_^2^ = 0.19) was specified for group differences on viewing different emotional facial expressions, based on a previous study conducted in our laboratory [20]. Within our chosen sample size and effect size, the power (1 – β) was approximately .90.

The study was approved by the ethical committee of the Department of Psychology of the University of Bologna and was conducted according to the principles of the Declaration of Helsinki. All the participants were fully informed about the scope and duration of the experimental procedure. Consent was obtained orally, in order to avoid identifiable record of consent and protect the privacy of persons participating in the research. The consent procedure was approved by the ethical committee of the Department of Psychology of the University of Bologna.

#### eVRT task

To test the ability of participants to remap others’ emotions onto their own body, we used an indirect task: Emotional Visual Remapping of Touch (eVRT, [10]).

*Materials*. Three human faces showing fearful, disgusted, and neutral facial expressions were chosen. Human faces were taken from the Pictures of Facial Affect dataset [54]. Short (3000 ms) videos were created in Microsoft Power Point that showed each face on a black background being either touched or approached by one or two human fingers. A computer running C.I.R.O software (http://www.cnc.unibo.psice.unibo/ciro) displayed the visual stimuli and collected responses. Electro-tactile stimulation was delivered via two constant current electrical stimulators (DS7A, Digitimer) connected to two pairs of electrodes (Neuroline, AMBU), one on each side of the participant’s face over the zygomatic arch.

*Procedure*. Following the staircase procedure used by Cardini and colleagues [10], the detection rate of electro-tactile stimulation was set to nearly 100% on one cheek and to approximately 60% on the other. The cheek that received stronger electro-tactile stimulation (left or right) was counterbalanced between participants. Confirming correct calibration, the mean detection rate of bilateral tactile stimulation across all experimental conditions was 53.02% (SEM = ±1.14%), and, when bilateral stimulation was not correctly identified, errors mostly consisted of reporting unilateral stimulation on the stronger side (M = 95.1% of errors, SEM = ±1.32%).

The experiment consisted of six blocks of VRT trials, two with neutral faces, two with fearful faces, and two with disgusted faces. Block order was counterbalanced between participants, and electro-tactile detection thresholds were re-calibrated between blocks. Each trial began with a face in the center of the screen and two fingers at the bottom of the screen, on either side of the chin. One or both of the fingers then moved upward and either touched the cheek on the same side of the screen, or touched a location about 5 cm lateral to the face before returning to the bottom of the screen. When the fingers reached the top of their trajectory (approximately 800 ms into the trial), electro-tactile stimulation was delivered to one or both of the participant’s cheeks. Participants used a keyboard to indicate whether they felt touch on the left cheek, on the right cheek, or on both cheeks. They were instructed to respond as quickly and accurately as possible, and informed that the location of apparent touch on the cheeks of the observed face was non-informative about the touch on their own face. Each trial combined one of two types of tactile stimulation (unilateral or bilateral), one of two types of visual stimulation (unilateral or bilateral), and one of two types of finger trajectories (touch or no-touch), resulting in 8 trial types that were repeated 12 times each block for a total of 96 trials per block, presented in a random order. Only trials with both bilateral tactile stimulation and bilateral finger movement (touch or no-touch) were analyzed.

#### Direct task

The direct emotion recognition task consisted of arousal and valence ratings of emotional facial expressions, as well as direct emotion recognition and labeling. The very same Ekman faces used in the eVRT were presented to participants: three different actors displaying fearful, disgusted and neutral expressions. Participants were asked to rate arousal and valence using a nine-point Likert scale, with 1 meaning “not very arousing” or “very negative emotion” and 9 meaning “highly arousing” or “very positive emotion.” Participants were also provided with a list of the six basic emotions, and they were required to label the emotion expressed in each Ekman face. Moreover, to assess whether the eVRT results could be related to individual differences in disgust sensitivity, the Disgust Scale (DS, [62]), a 32-item self-report questionnaire, was administered. The Disgust Scale is formed of two subscales that measure personal reactions to disgusting stimuli and personal evaluations of disgusting situations.

### Results

#### eVRT task

To investigate the effect of viewing emotional facial expressions on the VRT effect in alexithymia, the accuracy of HA and LA participants in identifying bilateral tactile stimulation was compared between trials in which both fingers touched or merely approached the observed face. We conducted an analysis of variance (ANOVA) on bilateral stimulation detection accuracy using Group (two levels: Low and High alexithymia) as a between-subjects variable, and Emotion (three levels: Fear, Disgust and Neutral) and Finger Trajectory (two levels: No-Touch and Touch) as within-subjects variables. Post-hoc comparisons (Duncan’s tests) were performed, when necessary, to compare single effects. The partial eta squared (η^2^_p_) was reported as an estimate of effect size [63]. The analysis revealed a significant main effect of Finger Trajectory (F[1,38] = 107.6; p<0.001; η_p_^2^ = 0.74). Moreover, the critical triple interaction Group x Emotion x Finger Trajectory was significant (F[2,76] = 14.7; p<0.001; η_p_^2^ = 0.28). Consequently, ANOVAs on bilateral stimulation detection accuracy using Emotion and Finger Trajectory as within-subjects variables were performed separately for the LA and HA groups.

In the LA group, the analysis revealed a significant main effect of Finger Trajectory (F[1,19] = 60; p<0.001; η_p_^2^ = 0.76). Moreover, the critical double interaction Emotion x Finger Trajectory was also significant (F[2,38] = 10.2; p = 0.001; η_p_^2^ = 0.35). Post-hoc tests showed that the Touch condition enhanced the accuracy of bilateral stimulation perception compared to the No-Touch condition for neutral faces (mean accuracy No-Touch: 57.7%, mean accuracy Touch: 69.2%, p = 0.002), fearful faces (57.6% vs. 78.4%, p<0.001) and disgusted faces (60.4% vs. 66.7%, p<0.001). In the Touch condition, the accuracy of bilateral stimulation detection was higher for fearful faces (78.4%) than for disgusted faces (66.7%, p<0.001) or neutral faces (69.2%, p<0.0001), and there was no difference between accuracy for neutral faces (69.2%) and disgusted faces (66.7%). Moreover, in the No-Touch condition there were no differences in the accuracy of bilateral stimulation detection between the three emotions: fear (57.6%), disgust (60.4%) and neutral (57.7%). This is important for ruling out the possibility that the results are due to increased attention to fearful or disgusted faces compared to neutral faces.

**Fig 1:**
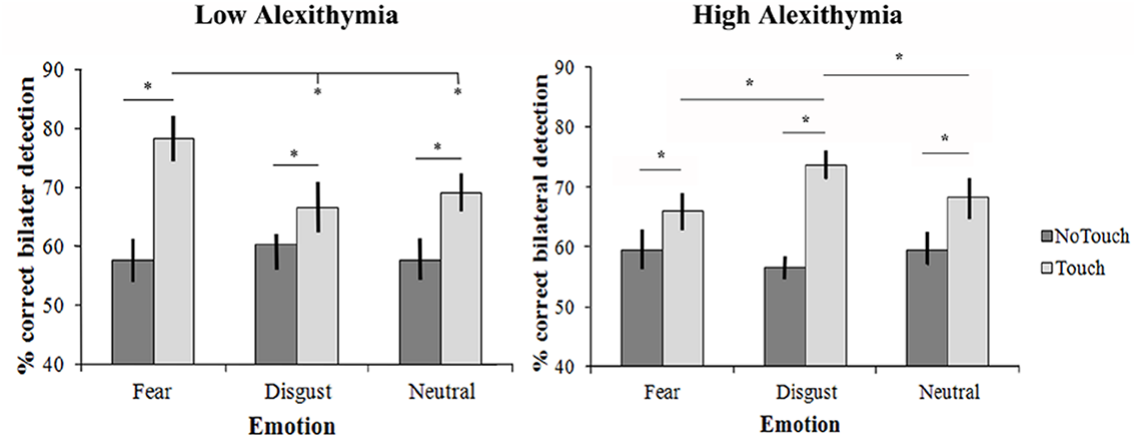
Results from the eVRT task in LA and HA participants, expressed as accuracy in detecting bilateral tactile stimulation while viewing movies showing either a fearful face, a disgusted face or a neutral face touched or just approached by two human fingers. Error bars show standard errors of the mean across LA and HA participants.

In the HA group, the analysis revealed a main effect of Finger Trajectory (F[1,19] = 47.7; p<0.001; η_p_^2^ = 0.72). Critically, the double interaction Emotion x Finger Trajectory was significant (F[2,38] = 5.7; p = 0.006; η_p_^2^ = 0.23). Post-hoc tests showed that the Touch condition enhanced the accuracy of bilateral stimulation perception compared to the No-Touch condition for neutral faces (mean accuracy no touch: 59.5%, mean accuracy touch: 68.3%, p<0.001), disgusted faces (56.6% vs 73.6%, p<0.001), and fearful faces (59.5% vs 66%, p = 0.007). In addition, in the Touch condition the accuracy of bilateral stimulation detection was higher for disgusted faces (73.6%) than for fearful (66%, p = 0.002) or neutral faces (68.3%, p = 0.02), but there was no difference between accuracy for neutral faces (68.3%) and fearful faces (66%). Moreover, in the No-Touch condition there were no differences in the accuracy of bilateral stimulation detection between the three emotions: fear (59.5%), disgust (56.6%), and neutral (59.5%). Again, this is important for ruling out the possibility that the results are due to increased attention to fearful or disgusted faces. Results are depicted in Fig 1.

**Fig 2:**
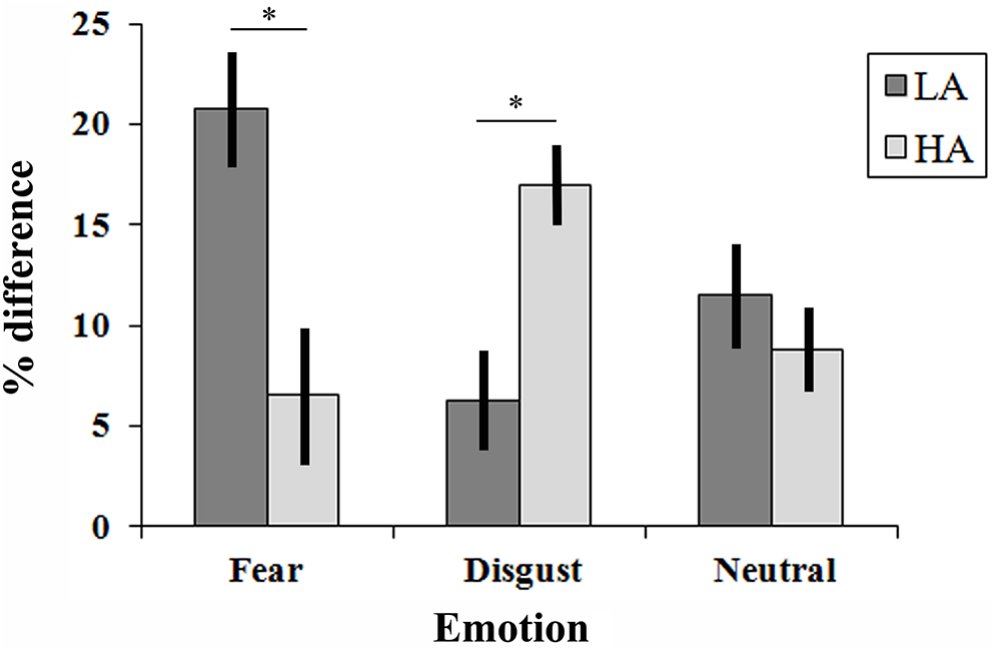
Results from the eVRT task in LA and HA participants, expressed as the difference in bilateral tactile detection accuracy between Touch and No-Touch conditions while viewing either a fearful face, a disgusted face or a neutral face. Error bars show standard errors of the mean across LA and HA participants.

To further explore the differences between the LA and HA groups in emotional modulation of tactile perception, an emotional VRT index was calculated by taking the difference between accuracy of bilateral stimulation detection in the No-Touch and Touch conditions (Touch–No-Touch). Emotional VRT indices for each emotion were compared in LA and HA participants. We performed an ANOVA on VRT indices using Group (two levels: LA and HA) as a between-subjects variable and Emotion (three levels: Fear, Disgust and Neutral) as a within-subjects variable. The analysis revealed a significant Group x Emotion interaction (F[2,76] = 14.7, p<0.001; η_p_^2^ = 0.28). Post-hoc tests in the LA group showed that the VRT index was larger (i.e. larger difference between No-Touch and Touch) when participants saw fearful faces (20.8%) than when they saw disgusted (6.3%, p = 0.001) or neutral faces (11.5%, p = 0.007), whereas there was no difference between neutral and disgusted expression conditions. On the contrary, in the HA group the VRT index was larger when participants saw disgusted faces (17%) than when they saw fearful (6.5%, p = 0.003) or neutral faces (8.8%, p = 0.01), whereas there was no difference between neutral and fearful expression conditions. The results are shown in Fig 2.

#### Direct task

To investigate the direct emotion recognition profile in alexithymia, LA and HA participants’ explicit arousal and valence ratings of the emotional facial expressions were compared. Moreover, participants were asked to directly name the emotion expressed by each face. Mean arousal and valence ratings and the number of emotion naming errors were analyzed using mixed factors ANOVAs with Group (two levels: LA and HA) as a between-subjects variable and Emotion (three levels: Fear, Disgust and Neutral) as a within-subjects variable.

With respect to arousal ratings, a significant main effect of Emotion (F[2,76] = 642.4; p<0.001; η_p_^2^ = 0.94) was found. Fearful (mean rate: 7.6) and disgusted (7.1) faces were rated as more arousing than neutral ones (1.6, p<0.001), and fearful faces were rated as more arousing than disgusted ones (p = 0.006). The main effect of Group and the interaction between Group and Emotion were not significant. With respect to valence ratings, the analysis revealed a significant effect of Emotion (F[2,76] = 193; p<0.001; η_p_^2^ = 0.84). Post-hoc tests revealed that valence ratings for fearful (mean rate: 2.4) and disgusted faces (2.3) were significantly lower than for neutral faces (4.7, p<0.001), whereas no difference was found between fearful and disgusted face ratings. The main effect of Group and the interaction between Group and Emotion were not significant. With respect to direct emotion recognition, a significant main effect of Group (F[1,38] = 9; p = 0.004; η_p_^2^ = 0.2) was found. HA participants made more errors in overall emotion identification compared to LA participants (0.98 and 0.58 errors, respectively, p = 0.004), but the interaction between Group and Emotion was not significant (F[2,76] = 1.12, p = 0.33; η_p_^2^ = 0.02).

Finally, to investigate whether eVRT findings could be related to individual differences in disgust sensitivity, LA and HA scores on the Disgust Scale and its subscales were compared by means of independent samples t-tests. LA and HA participants differed neither in their total DS score (mean 16.24 and 15.32 for LA and HA, respectively, t = 0.77, df = 38, p = 0.44, η_p_^2^ = 0.01), nor in their scores on the DS subscales (personal reaction subscale: 7.58 and 7.64 for LA and HA, respectively; t = -0.08, df = 38, p = 0.93, η_p_^2^ = 0.002; personal evaluation subscale: 8.59 and 7.74 for LA and HA, respectively; t = 1.25, df = 38, p = 0.21, η_p_^2^ = 0.05).

Altogether, these results suggest that the eVRT findings cannot be explained by differences in direct emotion recognition and/or differences in disgust sensitivity between the groups.

### Discussion

In keeping with previous results [50,51], the present findings confirm that viewing a face being touched enhances tactile perception on one’s own face, and that facial expressions of fear modulate this VRT effect [10] in LA but not in HA participants [20]. The new finding of the current study is that, unlike fearful expressions, expressions of disgust modulate the VRT effect in HA but not in LA participants.

This eVRT effect for disgust in HA cannot be explained in terms of a generic arousal effect induced by disgusted faces because the bilateral stimulation detection accuracy of the two groups did not differ in the No-Touch condition, nor could it result from differences in the perceived intensity of the emotional stimuli or sensitivity to disgusting stimuli between groups, because the HA participants’ arousal ratings and disgust sensitivity scores did not differ from those of the LA participants. The stronger VRT effect found in HA participants when the observed face showed a disgusted expression compared to a neutral or a fearful expression is in line with the physiological hypothesis, as stated in the Introduction. According to this hypothesis, the disgust-specific enhancement might reflect the different physiological responses induced by the experience of disgust as compared to fear.

Indeed, consistent with its apparent origin in defending against the ingestion of contaminated food [42,43], disgust is strongly associated with visceral changes, decreasing sensory interactions with the environment and redirecting one’s attention from the environment to internal bodily sensations reporting the physiological state of the body [64]. The eVRT findings of heightened disgust remapping in HA suggest that alexithymics might exhibit increased responses to stimuli producing heightened “physical” and visceral sensations, and that HA might mainly rely on “physical” information while performing the multisensory stimulation paradigm. Indeed, the “somatosensory amplification hypothesis” maintains that alexithymia is characterized by an amplification of the normal visceral phenomena accompanying emotional arousal, resulting in enhanced activation at the physiological levels and less activation in the cognitive-experiential domains [44–46,48,49]. In accordance with this hypothesis, and given that the emotion of disgust elicits strong visceral changes [41], it is possible to hypothesize that the previously described results of eVRT modulation by disgusted faces might be due to the higher ability of HA participants in perceiving their own internal bodily signals [44,65].

To address this possibility, the same participants of Experiment 1 underwent a second experiment, in which two indices of interoception ability were evaluated: interoceptive sensibility and interoceptive sensitivity, respectively. In line with Garfinkel and Critchley [66], interoceptive sensibility (ISb) refers to a dispositional tendency to be internally focused and was evaluated using the Bodily Perception Questionnaire (BPQ) [67]; while interoceptive sensitivity (ISt) refers to the objective accuracy in detecting internal bodily sensations and was evaluated using the Heartbeat Perception Task [68]. It was expected that HA would have higher ISb and ISt compared with LA participants [44,65].

Critically, the aim of this second experiment was to investigate a possible association between the eVRT results and the interoceptive abilities. To this aim, we first correlated the eVRT results with the interoceptive results, with the a priori prediction that the two interoception indices would correlate with the eVRT results for disgust; then we repeated the ANOVAs on the eVRT indices using ISb and ISt as covariates, with the a priori prediction that including the ISb or the ISt as a covariate would eliminate the association between alexithymia and eVRT. Both these expected results would suggest that the differences in eVRT between HA and LA groups were due to HA participants’ tendency to be focused on their own bodily signals [45,46,48,49].

## Experiment 2

In this second experiment, we tested whether LA and HA participants show different degrees of interoceptive sensibility (ISb) and sensitivity (ISt) and whether the degree of ISb and ISt correlate with eVRT effects.

### Material and Methods

#### Participants

The participants from Experiment 1 were asked to come back to participate in a second experiment. All 20 LA (5 male) and 20 HA (5 male) volunteers returned for the second experiment. This second study was approved by the ethical committee of the Department of Psychology of the University of Bologna and was conducted according to the principles of the Declaration of Helsinki. As for Experiment 1, consent for this second experiment was obtained orally, and this consent procedure was approved by the local ethical committee.

#### Interoceptive sensibility (ISb)

The Body Perception Questionnaire (BPQ) was adopted as a measure of interoceptive sensibility (ISb). The BPQ [67] is a 96-item self-report instrument that assesses body perception and interoceptive sensibility on four subscales: i) awareness subscale: participants report how aware they are of their bodily processes (e.g. swallowing frequently); ii) stress response: participants imagine being in a very stressful situation and rate their bodily changes due to that situation (e.g. emotional problems such as more frequent feelings of depression, frustration, rage or anger); iii) autonomic nervous system reactivity: participants respond to statements about their autonomic nervous system reactions (e.g. ‘my heart often beats irregularly’); iv) stress style subscale: participants evaluate the manner in which they respond to stress (e.g. ‘I have difficulty speaking’). Each item is rated on a five-point Likert scale ranging from 1 (never) to 5 (always). The higher the score, the stronger the participant’s perception of bodily sensations and interoceptive sensibility.

#### Interoceptive sensitivity (ISt)

The Heartbeat Perception Task was used as a measure of interoceptive sensitivity (ISt). For the heartbeat perception task, ECG measurements were taken using non-polarizable Ag-AgCl electrodes attached to the left and right wrists and referenced to the left mid-clavicle. Signals were recorded by a computer-based data acquisition system (Biopac MP150) and the corresponding software, AcqKnowledge (BIOPAC Systems Inc., Santa Barbara, CA). The heartbeat perception task was performed according to the Mental Tracking Method proposed by Schandry [68], using four intervals of 25, 35, 45, and 55 seconds. The four perception intervals were separated by standard resting periods (30 seconds). For all trials, participants were asked to silently count their heartbeats by concentrating on their heart activity. During heartbeat counting, participants were not permitted to take their pulse or to attempt any other physical manipulations that could facilitate the detection of heartbeats. Following the stop signal, participants were asked to verbally report the number of counted heartbeats. The participants were not informed about the lengths of the counting phases or about the quality of their performance. ISt was measured as a heartbeat perception score, calculated by taking the mean score across the four heartbeat perception intervals according to the following transformation: 1/4 Σ(1-(|recorded heartbeats—counted heartbeats|)/recorded heartbeats). The heartbeat perception score varies between 0 and 1. The maximum score of 1 indicates absolute accuracy of heartbeat perception. This heartbeat detection task is widely used to assess ISt [69,70], has good test-retest reliability (up to .81), and correlates highly with other heartbeat detection tasks [71].

### Results

#### Interoceptive sensibility (ISb)

Independent sample t-tests were conducted on the BPQ total and subscale scores of the HA and LA participants. The two groups significantly differed in total BPQ score (210.2 ± 43.9 and 262.3 ± 29.4 for LA and HA respectively, t = -4.4, df = 38, p<0.001, η_p_^2^ = 0.34). Moreover, scores on each of the four subscales differed between groups (awareness subscale: 107.4 ± 32.3 and 137.9 ± 16.7 for LA and HA respectively, t = -3.74, df = 38, p = 0.001, η_p_^2^ = 0.27; stress response: 26.7 ± 8.3 and 33.7 ± 6.6 for LA and HA respectively, t = -2.96, df = 38, p = 0.005, η_p_^2^ = 0.19; autonomic nervous system reactivity: 46.4 ± 9.3 and 57 ± 13.3 for LA and HA respectively, t = -2.91, df = 38, p = 0.006, η_p_^2^ = 0.18; stress style subscale: 29.7 ± 4.2 and 33.6 ± 5.1 for LA and HA respectively, t = -2.64, df = 38, p = 0.012, η_p_^2^ = 0.16). After correction for multiple comparisons, the differences between groups on the BPQ total score and the awareness subscale were still significant. Overall, those results reveal higher interoceptive sensibility in HA compared to LA participants.

#### Interoceptive sensitivity (ISt)

Independent samples t-tests were conducted on the ISt indices (heartbeat perception scores) of the LA and HA groups. The two groups significantly differed in heartbeat perception ability (0.63 ± 0.16 and 0.92 ± 0.05 for LA and HA respectively, t = -7.7, df = 38, p<0.001, η_p_^2^ = 0.61), with HA participants showing higher ISt than LA participants.

#### Link between ISb and eVRT performance

To investigate the link between ISb and eVRT, Spearman correlations were performed across groups between eVRT indices for the three emotions (neutral, fear and disgust) and the ISb as measured by the total BPQ score. No correlations were significant (eVRT for fear and ISb: r = -0.26, p = 0.1; eVRT for disgust and ISb: r = 0.21, p = 0.18;; eVRT for neutral and ISb: r = -0.14, p = 0.37).

#### Link between ISt and VRT performance

**Fig 3:**
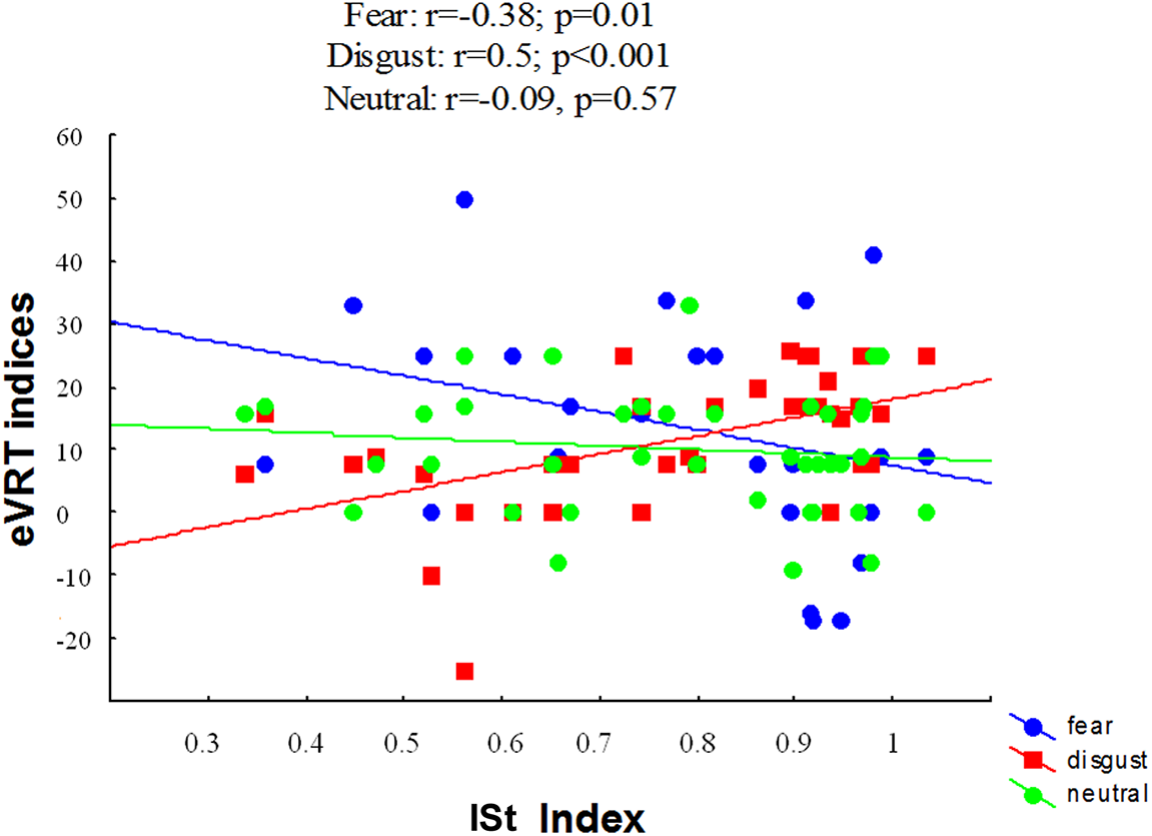
Correlations between eVRT indices for fearful, disgusted and neutral expressions and the ISt index (heartbeat perception score).

To investigate the link between ISt and eVRT, Spearman correlations between eVRT indices for the three emotions (neutral, fear and disgust) and the ISt index (heartbeat perception score) were performed across groups. There was a negative correlation between ISt and eVRT for fear (r = -0.38, p = 0.01), but a positive correlation between ISt and eVRT for disgust (r = 0.5, p<0.001). No correlation was found between ISt and eVRT for neutral faces (r = -0.09, p = 0.57). After correcting for multiple comparisons, the correlation between ISt and disgust and ISt and fear were still significant. In other words, the higher the ISt score, the bigger the eVRT index for disgust and the lower the eVRT for fear. This suggests that the eVRT effect for disgust and fear were linked to interoceptive signals. Results are illustrated in Fig 3.

Furthermore, according to our hypothesis that ISt should predict the eVRT disgust effect in alexithymia, we added ISt index (heartbeat perception score) as a covariate to the ANOVA on bilateral stimuli detection accuracy with Group as a between-subjects variable and Emotion and Finger Trajectory as a within-subjects variables. With ISt as a covariate, the main effect of Finger Trajectory was no more significant (F[2,37] = 0.28; p = 0.59; η_p_^2^ = 0.007). Although the Group x Emotion x Finger Trajectory interaction was still significant (F[2,74] = 4.01; p = 0.02; η_p_^2^ = 0.1), when analyzing each alexithymia group individually, the interaction Emotion x Finger Trajectory was not significant neither in the LA (F[2,36] = 0.99; p = 0.38; η_p_^2^ = 0.05) nor in the HA (F[2,36] = 0.52; p = 0.59; η_p_^2^ = 0.02) group. This result indicates that the differences in eVRT between HA and LA groups were linked to the HA participants’ heightened focus on their own body signals.

### Discussion

The critical findings of the second experiment were that the interoceptive sensitivity correlates with the eVRT effect for disgusted faces, and that, if the influence of the interoceptive sensititivity were removed from the eVRT analysis, the eVRT effect for disgusted faces was no longer present. Importantly, the significant association between ISt and the eVRT effect for disgust suggests that the greater the ability to perceive visceral (i.e. cardiovascular) signals, the stronger the remapping of others’ disgusted expressions onto oneself. By contrast, no correlation was found between ISt and the eVRT indices for fearful and neutral expressions. These results indicate that the eVRT findings for disgust are strictly associated with the interoceptive abilities of participants, i.e. with their tendency to be focused on their own internal bodily signals and, as a consequence, being more able to detect them. This results support the hypothesis that HA might rely on “physical” and internal bodily information while performing the multisensory stimulation paradigm, since they exhibit increased responses to stimuli producing heightened “physical” and visceral sensations [72]. As such, these results accord with our physiological hypothesis, suggesting that the physiological underpinnings of disgust may play a crucial role in modulating the activity of somatosensory cortices during eVRT with disgusted faces.

Another result of this second experiment is that HA participants showed higher interoception, both ISb and ISt, than LA participants. These findings are in line with recent work using BPQ (ISb) [65], but not with other results from the heartbeat perception task (ISt) [73]. However, the discrepancy between the previous heartbeat perception study and the present results might be due to methodological differences, for instance, in the participant inclusion procedure. Indeed, in Herbert et al.’s [73] study only the TAS-20 has been used, whereas in the present study we adopted an highly selective procedure, involving the TAS-20, a semi-structured interview (DCPR) and excluding participants with clinical depression. As a consequence, the present study has the advantage of a higher specificity in participant selection. Moreover, the statistical analyses conducted were different as well. Contrarily to our study, in which we directly compared participants scoring in the upper and lower quartile of TAS-20, respectively, Herbert et al. [73] included all participants, regardless of their TAS-20 score, and performed a regression analysis between TAS-20 score and heartbeat perception task. As a consequence, their participants TAS score had a small variance, since relatively few HA individuals had been included.

## General Discussion

The present study aimed at understanding whether the emotion embodiment difficulties we previously observed in alexithymic individuals when viewing fearful faces [20] were also present during the observation of disgusted faces. Results revealed a clear dissociation between the remapping of fearful and disgusted expressions in low and high alexithymic participants: while the emotion of fear is remapped in LA but not in HA participants, the opposite pattern is true for the emotion of disgust.

In LA participants, the remapping of fearful expressions replicates the findings of previous studies that used the same paradigm in normal populations [10], and in participants with low level of alexithymia [20], namely an enhancement of the VRT effect when participants viewed fearful expressions. This effect might reflect a fear-related pre-activation of the fronto-parietal network and related somatosensory cortices involved in the VRT mechanism [8]. The absence of enhanced VRT when LA participants viewed disgusted expressions, relative to neutral expressions, further corroborates the previous hypothesis that the emotion-related enhancement of the VRT effect is specific to fearful expressions in non-alexithymic individuals [10], since this enhancement is not elicited by positive emotional expressions (i.e. happiness [10,20]), or by other negative emotional expressions, such as anger [10], and disgust (present results).

The results of eVRT with fearful expressions in HA participants replicate those of our previous study using the same paradigm in alexithymia [20]: the expected enhancement of the VRT effect when viewing fearful faces is absent in HA participants. This result has been interpreted as an inability of HA participants to remap the fearful expressions of others onto their own somatosensory system. This is probably due to a hypo-activation of the amygdala in alexithymia [74], which might fail to pre-activate somatosensory cortices. Preliminary findings from patients with amygdala lesions support this hypothesis, as these patients do not show the typical enhancement of VRT when viewing fearful expressions, whereas patients with spared amygdalae and damage to other brain regions show a normal eVRT effect for fear [75].

Critically, the present study shows that alexithymic participants have an abnormally strong response to disgust, manifested as enhanced disgust remapping compared to LA participants. Since alexithymic individual are mainly focused on their own, emotion-provoked, physical states, rather than on their subjective experience of emotion [45, 46, 48, 76], our physiological hypothesis was that HA participants might show an enhancement of the VRT effect for emotional stimuli that are accompanied by strong physical and visceral sensations, i.e. disgust. Moreover, we hypothesized that the enhanced modulation of eVRT by disgusted faces might be related to the higher ability of HA participants in perceiving and reporting their own bodily signals [44,65]. In line with this hypothesis, in a second experiment we found that the interoceptive abilities of HA participants were tightly linked to the eVRT performance for disgusted faces, such that the the greater the ability to perceive bodily (i.e. cardiovascular) signals, the stronger the remapping of others’ disgusted expressions onto oneself.

Previous findings showed that environmental stimuli trigger emotional responses that are associated with an impoverished conscious experience of emotion in HA individuals [12, 74, 77]. Instead, HA individuals exhibit the tendency to experience physical sensations in response to emotion-provoking stimuli [44,49], thereby suggesting a different mode of emotion processing in alexithymia. The present data suggest that this phenomenon might be more pronounced for emotions associated with stronger bodily changes, namely disgust, thereby explaining the higher eVRT for disgust in HA subjects observed here.

Our results have crucial implications for alexithymia research, since they reveal that negative emotions are abnormally embodied in HA individuals, but with important differences between emotions. Indeed, while the embodiment of the emotion of fear is defective (see also [20]), the embodiment of the emotion of disgust is heightened. Previous research has demonstrated that HA subjects may perceive signals from the body in an aberrant manner, and may be more aroused by interoception of unpleasant stimuli than LA individuals (see [16], for a review). Consistent with these studies, and with the somatosensory amplification hypothesis of alexithymia [46,48, 49], the present results indicate that alexithymic individuals manifest an abnormal embodiment of disgust, a basic emotion characterized by greater interoceptive and physiological arousal [41,42].

Previous studies suggest that the VRT effect is evoked by means of a pre-activation of somatosensory cortices when viewing touch on others’ face [8,10]. Likewise, the abnormal eVRT effect while observing disgusted faces may be induced by an enhancement of somatosensory cortices activation. Indeed, the emotion of disgust has been found to preferentially activate somatosensory and insular cortices [37–40,78]., Previous neuroimaging findings in alexithymia research have shown increased insular and somatosensory reactivity in response to negative stimuli in HA participants [44,79,80]. Thus, the strict relation between the insula and the somatosensory cortices, and their greater activation in alexithymia can account for how the disgusted faces may increase VRT effect in HA, but not LA, participants. Interestingly, a recent work showed that both alexithymia and heightened interoceptive abilities are closely associated with increased activity of the insula [65], thereby suggesting that this brain region may be crucially implicated in the interconnection between increased perception of bodily responses and alexithymic features. Thus, previous and present findings, converge to support the hypothesis that people with HA are more likely to be focused on their bodily states and physical sensations than people with LA [44, 45, 65], possibly due to aberrant insular and somatosensory activation in responses to emotion-evoking events [44,79, 80].

The current results of higher interoceptive abilities in alexithymia appear to be prima facie inconsistent with previous studies indicating that subjective emotional experience (which is impoverished in alexithymia) and interoceptive accuracy are strictly interdependent [81–84], and that the same neural regions, particularly the anterior insula, are involved in appraisal of emotions and bodily physiology [85–89]. However, although strongly linked, the precise contribution of interoceptive accuracy and physiological responses to conscious experience of emotion still remains controversial [90,91]. In fact, interoceptive accuracy can be necessary but not sufficient for the conscious experience of emotions. For instance, according to the theoretical construct of emotion proposed by Lane and Schwartz [92], emotional awareness can be graded in different ‘levels’, and awareness of physiological responses are graded in the lower level. In this view, people with alexithymia rely on a lower, physiological level of emotional awareness, thereby stagnating at the level of bodily sensations. Although being able to detect their own visceral changes, alexithymic participants may fail to link these visceral signals to higher levels of emotional processing. In this way, the emotion-evoking event is perceived only at the “physical” level, devoid of any emotional implication. Thus, our results did not contradict, but rather expand, previous literature, supporting the hypothesis of dissociation between interoceptive accuracy and emotional awareness in alexithymia.

As a final observation, alexithymia is a risk factor for the so called “pathologies of the disgust system”, i.e. pathologies in which the responses to disgusting stimuli are disproportionate to actual risk [93], such as the obsessive compulsive disorders (contamination obsessions) [94], eating disorder (disgust for the bodily self; [95,96]), some symptoms of schizophrenia [97,98] and hypochondriasis [99,100]. The current results suggest that HA individuals are abnormally focused on their visceral reactions, thus suggesting possible mechanisms increasing the risk to develop the pathologies of the disgust system.

To summarize, the present findings highlight a dissociation between the remapping of fear and disgust in people with HA and suggest that the heighened abilities of HA, compared with LA, participants in embodying the emotion of disgust could be associated with their predisposition to abnormally detect internal bodily sensations. We suggest that the emotional profile of HA individuals on the eVRT task could be related to their abnormal predisposition to be focalized on their internal bodily signals reporting the body’s physiological state, and to their tendency to experience emotions in a “physical” way. Finally, we speculated that these results in HA could be due to an enhancement of insular activity during the perception of disgusted faces.
